# Stressing the importance of plant specialized metabolites: omics-based approaches for discovering specialized metabolism in plant stress responses

**DOI:** 10.3389/fpls.2023.1272363

**Published:** 2023-11-08

**Authors:** Mengxi Wu, Trent R. Northen, Yezhang Ding

**Affiliations:** ^1^ College of Landscape Architecture, Sichuan Agricultural University, Chengdu, China; ^2^ Joint Genome Institute, Lawrence Berkeley National Laboratory, Berkeley, CA, United States; ^3^ Environmental Genomics and Systems Biology Division, Lawrence Berkeley National Laboratory, Berkeley, CA, United States

**Keywords:** metabolomics, multi-omics, plant specialized metabolites, biosynthesis, biotic and abiotic stress

## Abstract

Plants produce a diverse range of specialized metabolites that play pivotal roles in mediating environmental interactions and stress adaptation. These unique chemical compounds also hold significant agricultural, medicinal, and industrial values. Despite the expanding knowledge of their functions in plant stress interactions, understanding the intricate biosynthetic pathways of these natural products remains challenging due to gene and pathway redundancy, multifunctionality of proteins, and the activity of enzymes with broad substrate specificity. In the past decade, substantial progress in genomics, transcriptomics, metabolomics, and proteomics has made the exploration of plant specialized metabolism more feasible than ever before. Notably, recent advances in integrative multi-omics and computational approaches, along with other technologies, are accelerating the discovery of plant specialized metabolism. In this review, we present a summary of the recent progress in the discovery of plant stress-related specialized metabolites. Emphasis is placed on the application of advanced omics-based approaches and other techniques in studying plant stress-related specialized metabolism. Additionally, we discuss the high-throughput methods for gene functional characterization. These advances hold great promise for harnessing the potential of specialized metabolites to enhance plant stress resilience in the future.

## 1 Introduction

In recent years, climate change, anthropogenic activities, and natural resource depletion have emerged as critical global threats to agriculture (Zhao et al., 2017; Fadiji et al., 2021). Climate change has engendered severe abiotic stresses such as salinity, drought, and extremely high and low temperatures (Fadiji et al., 2021), which pose a significant threat and drastically reduce plant productivity. It has been estimated that with every 1°C increase in the world’s average temperature, plants, such as maize (*Zea mays*), Sorghum (*Sorghum bicolor*), wheat (*Triticum aestivum*), rice (*Oryza sativa*), and soybean (*Glycine max*), experienced yield losses 3- 8% over 29 years of warming trends (Zhao et al., 2017). Particularly, drought and salinity caused by climate change pose a threat to approximately 50% of the global cultivated and irrigated agricultural land (Orimoloye, 2022; Singh, 2022). Climate change not only imposes abiotic stress on plants but also exacerbates the occurrence of biotic factors, such as bacteria, fungi, herbivores, and insects. Research has shown that up to 40% of crop production is affected by pests and diseases that are exacerbated by climate change (Savary et al., 2019). Given these limiting factors, scientists are continuously making efforts to search for novel, safe, and environmentally friendly approaches to enhance plant performance under stress conditions, including those that harness plant specialized metabolites to mitigate biotic and abiotic stresses.

Extensive research has suggested that each biotic and abiotic stress perceived by plants triggers systemic signaling and acclimation responses, leading to the accumulation of specialized metabolites (Marone et al., 2022). Despite the significant energy expenditure involved in their production, these specialized compounds provide plants with an effective defense mechanism to cope with biotic and abiotic stress challenges, like protecting plants against herbivores, insects, and pathogens, as well as mitigating the adverse effects of environmental factors (D'Amelia et al., 2021; Ding et al., 2021b; Marone et al., 2022). Meanwhile, these unique defensive compounds have wide-ranging applications in industries such as food, pharmaceuticals, and chemicals, owing to their nutritional and therapeutic values. For example, artemisinin, a well-known sesquiterpenoid produced by *Artemisia annua*, has been widely utilized in the treatment of malaria, a life-threatening parasitic disease caused by *Plasmodium* parasites (Chen et al., 2021). Accordingly, understanding the genetic basis of specialized metabolite biosynthesis and their ecological functions will contribute to fully exploring the potential of these natural products and enable the innovation of novel strategies to improve plant stress resilience.

Undoubtedly, the advancement of analytical chemistry has equipped diverse research groups with the capability to explore the existence of both unknown and known plant specialized metabolites as traits in various biological investigations. However, specialized metabolites are typically restricted to specific plant populations or lineages, presenting challenges in determining their exact roles in ecological interactions and understanding the genetic mechanisms responsible for their biosynthesis and accumulation (D'Amelia et al., 2021). Over the last decade, these limitations have been increasingly overcome through the rapid expansion of omics technologies, including metabolomics, genomics, transcriptomics, and proteomics (Ding et al., 2019; Ding et al., 2020; Jacobowitz and Weng, 2020; Ding et al., 2021b). While previous reviews have covered various aspects of plant specialized metabolism (Fang and Luo, 2019; Jacobowitz and Weng, 2020; D'Amelia et al., 2021; Ding et al., 2021b; Singh, 2022), it was necessary to provide an overview on the most recent research and advanced methodologies for studying plant specialized metabolism, particularly in the context of plant stress responses. Here, we review the recent advancements in the field of plant specialized metabolism and discuss the application of omics-based approaches to study the genetic mechanisms underlying the biosynthesis, accumulation, and biological functions of plant stress-related specialized metabolites.

## 2 Biological roles of specialized metabolites in plant stress responses

**Figure 1:**
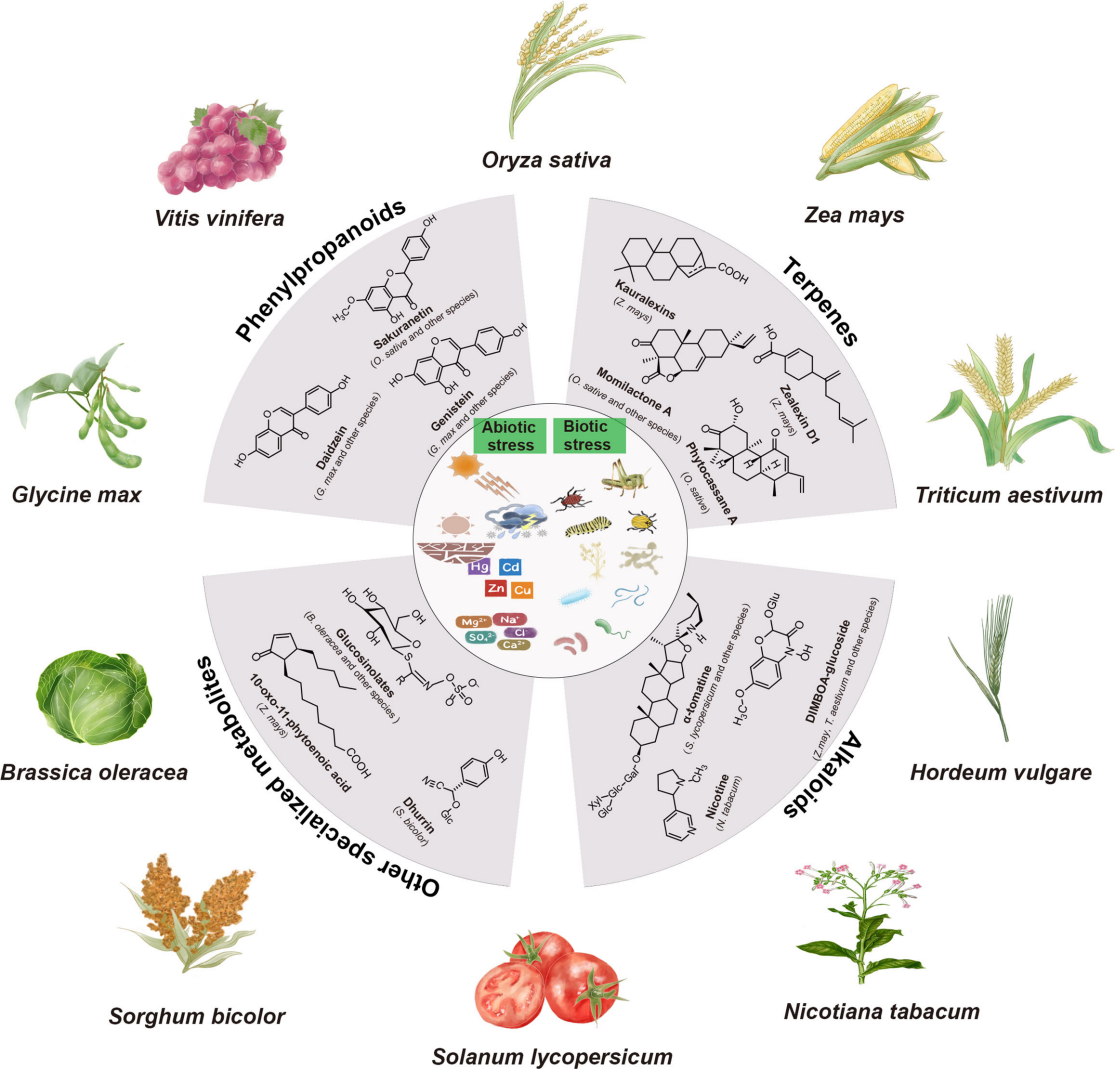
Major classes of plant specialized metabolites and their biological functions. The major classes of plant specialized metabolites, including phenylpropanoids, terpenes, alkaloids, and other specialized metabolites are displayed. Specialized metabolites play crucial roles in protecting plants against both abiotic stresses (e.g., light, heat, drought, cold, flood, salinity, and metals) and biotic stresses (e.g., pests and pathogens).

Plant specialized metabolites play crucial roles in various physiological processes, such as plant growth, development, and response to diverse biotic and abiotic stress (Marone et al., 2022). Differing from primary metabolites, specialized metabolites are typically produced in response to specific environmental stimuli or other signaling cues, as well as during specific developmental stages (Jacobowitz and Weng, 2020; Garagounis et al., 2021). When plants face adverse growth conditions, the production of various specialized metabolites enhances their chances of survival (**Figure 1**).

One of the prominent functions of specialized metabolites in plants is to act as a defense mechanism against biotic stressors, such as pathogens, herbivores, and other pests.

Defensive phytochemical specialized metabolites can be categorized into two groups: phytoanticipins and phytoalexins (VanEtten et al., 1994; Piasecka et al., 2015). Phytoanticipins are constitutively present or synthesized from preexisting precursors (VanEtten et al., 1994). Notable examples of phytoanticipins include saponins, cyanogenic glucoside, glucosinolates, and benzoxazinone glucosides. For instance, α-tomatine, a major saponin in tomato (*Solanum lycopersicum*), has the capability to induce programmed cell death in fungi (Piasecka et al., 2015). Dhurrin,a cyanogenic glucosides present in sorghum (*Sorghum bicolor*), can undergo degradation, leading to the release of toxic cyanide, thereby deterring pests (Laursen et al., 2016). In contrast, phytoalexins are synthesized *de novo* when plants detect a pathogen or pest (Piasecka et al., 2015). Non-volatile terpenoids are well-documented and fascinating examples of phytoalexins (Schmelz et al., 2014). In maize, diterpenoid phytoalexins like dolabralexins and kauralexins, as well as sesquiterpenoid phytoalexins such as α/β-costic acids and zealexins, have been identified as part of the maize’s defense response against fungal infections (Ding et al., 2017; Mafu et al., 2018; Ding et al., 2019; Ding et al., 2020). Likewise, rice plants are capable of producing various diterpenoid phytoalexins, known as momilactones, phytocassanes, and oryzalexins, which have been shown to contribute to the rice’s stable resistance against major fungal diseases (Wang et al., 2012; Schmelz et al., 2014). Additionally, other classes of specialized metabolites, such as benzoxazinoids and flavonoids, have also been reported to play similar defensive roles (Valletta et al., 2023; Singh et al., 2023a). A rice-flavanone-type phytoalexin, namely sakuranetin, is one such example, which inhibits the germination of the conidia of fungal pathogens (Hasegawa et al., 2014).

Furthermore, it is increasingly evident that plants employ specialized metabolites to attract symbiotic bacteria and arbuscular mycorrhizal fungi, as well as shape microbiomes in the rhizosphere and phyllosphere (Sasse et al., 2018; Garagounis et al., 2021; Singh et al., 2023a). Among the well-studied models are the interactions between legumes and their rhizosphere bacteria. The roots of legume plants release specialized metabolites such as isoflavones and saponins into the rhizosphere as signaling compounds to attract symbiotic bacteria, such as *Azorhizobium*, *Rhizobium*, and *Pararhizobium* (Pang et al., 2021). In addition, many root-derived specialized metabolites have been shown to have impacts on rhizosphere microbial compositions. For example, a recent study revealed that daidzein, a specific isoflavone secreted from soybean roots, plays a role in regulating the assembly of bacterial communities in the rhizosphere (Okutani et al., 2020).

Specialized metabolites in plants also serve another important function: assisting plants in alleviating stresses caused by abiotic factors, such as extreme temperatures, drought, salinity, and ultraviolet radiation. Under abiotic stress, plants generate harmful reactive oxygen species (ROS), such as singlet oxygen (O_2_), reactive superoxide anion radical (O_2_^•−^), hydrogen peroxide (H_2_O_2_), and hydroxyl radical (•OH) (Agati and Tattini, 2010; Barnes et al., 2016; Piasecka et al., 2017). Disruption of the balance between ROS generation and endogenous antioxidant defense mechanisms results in oxidative stress (Chan et al., 2016). In cases where the production of antioxidant enzymes is insufficient to counteract the level of oxidation, specialized metabolites with antioxidant activity become a vital tool in buffering ROS accumulation, mainly flavonoids and phenolic compounds (Agati and Tattini, 2010; Nakabayashi et al., 2014; Barnes et al., 2016). The UV-B-responsive flavonoids function as quenchers of ROS involved in the UV-protection mechanism (Agati and Tattini, 2010; Barnes et al., 2016). The excessive accumulation of flavonoids with antioxidative properties has been found to enhance drought stress tolerance in maize (Li et al., 2021). Additionally, specialized metabolites with antioxidant activity can also provide protection against biotic stress. For instance, metabolic engineering of antioxidative pigments, like anthocyanins and betalains, can enhance plant resistance against the necrotrophic fungal pathogen, *Botrytis cinerea* (Zhang et al., 2013; Polturak et al., 2017).

## 3 Major classes of plant specialized metabolites

Plant specialized metabolites exhibit remarkable structural diversity surpassing that of primary metabolites, with many originating from primary metabolic precursors (Ding et al., 2021b). The exact number of plant specialized metabolites remains unknown, but it has been estimated to range from 200,000 to 1,000,000 (Dixon and Strack, 2003; Afendi et al., 2012). Here, we present a concise overview of the major classes of specialized metabolites involved in plant-abiotic and biotic interactions (**Figure 1**).

### 3.1 Phenylpropanoids

Phenylpropanoids consist of a phenyl ring and a three-carbon side chain, which are derived from phenylalanine through the shikimic acid pathway (Agati and Tattini, 2010; Vogt, 2010). The diverse substituents on the benzene ring and the position of the propenyl double bond, lead to the generation of a wide range of compounds with various biological activities (Dong and Lin, 2021). The general phenylpropanoid pathway involves three key enzymes: phenylalanine ammonia-lyase (PAL), cinnamate 4-hydroxylase (C4H), and 4-coumarate-CoA ligase (4CL), which provide precursors for the synthesis of flavonoids and lignin (Agati and Tattini, 2010; Dong and Lin, 2021). Lignin polymers are typically composed of three fundamental monolignols: *p*-hydroxyphenyl (H), guaiacyl (G), and syringyl (S), which are derived from *p*-coumaryl alcohols, coniferyl alcohols, and sinapyl alcohols, respectively. The most recent advancements in the lignin biosynthetic pathways and how flux through the pathway is regulated in plants have been comprehensively reviewed (Vanholme et al., 2019; Yao et al., 2021).

#### 3.1.1 Flavonoids

Flavonoid metabolism is another important branch of phenylpropanoid metabolism, and research has identified over 8,000 different flavonoid compounds to date (Shomali et al., 2022). Flavonoids can act as antioxidants, signal molecules, pigments, phytoalexins, and detoxifying agents (Agati and Tattini, 2010; Barnes et al., 2016; Zhang et al., 2023). Moreover, flavonoids possess numerous medicinal benefits, including anti-inflammatory, antidiabetic, anticancer, and antiviral properties (Dias et al., 2021; Shomali et al., 2022).

Almost all flavonoids possess a C6-C3-C6 structural backbone, which consists of two benzene rings with phenolic hydroxyl groups (A and B rings) connected to a three-carbon pyran ring (C) (Dias et al., 2021). The core skeleton of the flavonoid biosynthetic pathway has been extensively studied in terms of the biochemical, molecular, and genetic mechanisms of the enzymes involved. This synthesis involves two primary pathways: the phenylpropanoid pathway, which generates the phenyl propanoid (C6-C3) skeleton, and the polyketide pathway, which provides the building blocks for polymerized C2 units (Dias et al., 2021; Shomali et al., 2022). The naturally occurring basic skeleton of C6-C3-C6 commonly undergoes various enzymatic modifications, including hydroxylation, glycosylation, methylation, and acylation (Shomali et al., 2022; Liu et al., 2022b). Based on the oxidation level or the substitution patterns of the middle C-ring, flavonoids can be classified into six major sub-classes: flavonols, flavones, isoflavones, flavanones, flavan-3-ols, and anthocyanins (Tohge et al., 2018; Shomali et al., 2022; Liu et al., 2022b).

Chalcone synthase (CHS) initiates the synthesis by utilizing malonyl-CoA molecules from the polyketide pathway and *p-*coumaroyl CoA from the phenylpropanoid pathway to produce naringenin chalcone, which is then converted into flavanone naringenin by chalcone isomerase (CHI) (Tohge et al., 2018; Dias et al., 2021). Flavanone naringenin serves as a biochemical precursor in the biosynthesis of other flavonoids, such as flavones, flavonols and anthocyanins (Tohge et al., 2018; Liu et al., 2021). Basic hydroxylation is a common occurrence in naringenin at positions C4’, C5, and C7, while additional hydroxyl groups can also be found at positions C3’, C3, C5’, C6, and C8 (Liu et al., 2022b). Hydroxylases play an important role in the biosynthesis of hydroxylated flavonoids. Flavanone 3-hydroxylase (F3H) is a key enzyme for the hydroxylation of the C ring, converting naringenin into dihydroquercetin, which further contributes to the biosynthesis of flavonols and anthocyanidins (Lara et al., 2020). Overexpression of *SbF3H1* in sorghum deficient in 3-hydroxylated flavonoids redirects carbon flow towards the production of 3-hydroxylated flavonoids, leading to an enriched flavonoid profile in various tissues, potentially enhancing defense response and improving the nutraceutical value of sorghum grain/bran (Wang et al., 2020). Flavonoid 3’-hydroxylase (F3’H) and flavonoid 3’,5’-hydroxylase (F3’5’H) play crucial roles as enzymes facilitating the hydroxylation of the B ring. Dihydrokaempferol can be further catalyzed by F3’H and F3’5’H, respectively, resulting in the formation of either dihydroquercetin or dihydromyricetin. Subsequently, dihydroflavonol reductase (DFR), an enzyme relying on NADPH, facilitates the reduction of dihydroflavonols such as dihydroquercetin and dihydromyricetin, resulting in the production of colorless anthocyanins. These colorless anthocyanins are then converted into colored anthocyanins through anthocyanidin synthase (ANS) catalysis before being transformed into stable anthocyanins (Liu et al., 2021).

In addition, flavone synthase (FNS) enzymes, including two distinct types known as FNS-I and FNS-II, are responsible for catalyzing the conversion of flavanones into flavones. FNS-I belongs to the Fe^2+^/2-oxoglutarate-dependent dioxygenase (2-OGDD) family. Previous studies have identified OsFNS in rice and ZmFNSI-1 in maize as FNS-I enzymes that catalyze the conversion of naringenin to apigenin, a major plant flavone (Kim et al., 2008; Falcone Ferreyra et al., 2015). On the other hand, FNS-II is a member of cytochrome P450 enzymes derived from the CYP93B subfamily in dicots and the CYP93G subfamily in monocots (Lam et al., 2014; Lam et al., 2017). In rice, OsCYP93G2 converts eriodictyol and naringenin into the corresponding 2-hydroxyflavanones, which are essential components required for the biosynthesis of C-glycosylflavones (Du et al., 2010). In the monocot family *Poaceae*, tricin, a notably prevalent flavonoid form, is commonly observed as an *O*-linked conjugate in vegetative tissues. The biosynthesis of tricin conjugates involves the conversion of naringenin to apigenin by FNSII, followed by sequential hydroxylation and *O*-methylation of tricin to generate various downstream tricin derivatives (Lam et al., 2017).

Besides hydroxylation, glycosylation is commonly found in flavonoids. Glycosylated anthocyanidins are a common type of flavonoid derivatives responsible for the colors in most flowers and fruits (Rinaldo et al., 2015). In dicots crops, *O*-glycosylated flavonols/isoflavones are predominantly accumulated as the major type of flavonoids, while monocot crops primarily produce *C*-glycosylated flavones (Tohge et al., 2018). *O*-glycosyltransferases utilize oxygen to link the sugar moiety to the flavonoid skeleton in *O-*glycosyl flavones, whereas the glucose moiety in *C*-glycosyl flavones directly binds to the flavone backbone (Funaki et al., 2015; Sun et al., 2022). For instance, in soybean, daidzein (4’,7-dihydroxyisoflavone) and genistein (4’,5,7-trihydroxyisoflavone) undergo enzymatically glycosylated by 7-*O*-glycosyltransferase, resulting in the production of genistin and daidzin, respectively (Funaki et al., 2015). In rice and maize, *C*-glucosyltransferases, including OsCGT, ZmUGT708A6, and ZmCGT1, catalyze flavone *C*-glycosylation at either the C-8 or C-6 position of 2-hydroxyflavanone, leading to the formation of flavone-*C*-glycosides after dehydration (Brazier-Hicks et al., 2009; Sun et al., 2022). The flavone glycosides, especially *C*/*O*- glycosyl flavones, play a positive role in plant UV-B protection (Brazier-Hicks et al., 2009; Peng et al., 2017). More importantly, *C*-glycosyl flavones have been shown to potentially enhance crops responses to abiotic and biotic stress like nitrogen limitation (Zhang et al., 2017), defense against pests (Casas et al., 2014), and fungal diseases (McNally et al., 2003).

#### 3.1.2 Hydroxycinnamate amides

Other phenylpropanoid metabolites include hydroxycinnamate amides (HCAAs), phenylpropanoid esters, lignans, and sporopollenin (Agati and Tattini, 2010; Vogt, 2010). HCAAs, alternatively known as phenylamides or phenolamides, are also a broad array of plant specialized phenylpropanoid metabolites, serving important roles in stress tolerance (Liu et al., 2022a). In particular, the accumulation of HCAAs in plants has been linked to enhanced resistance against various plant pathogens (Muroi et al., 2009; Seybold et al., 2020; Ding et al., 2021b). These HCAAs are synthesized through the conjugation of hydroxycinnamic acids (HCAs) such as cinnamic, *p-*coumaric, caffeic, ferulic, and benzoic acids with amines such as serotonin, tryptamine, putrescine, and agmatine (Zeiss et al., 2021). Recent studies have identified several HCAAs that function as phytoalexins in Poaceae. For instance, in rice, these HCAAs exhibited inducibility and antimicrobial activity against the pathogen *X. oryzae* (Morimoto et al., 2018). In barley (*Hordeum vulgare*), the accumulation of HCAAs, specifically 9-hydroxy-8-oxotryptamine and 8-oxotryptamine, has been observed in response to *Fusarium* infection, which are synthesized through the oxidation of *N*-cinnamoyl tryptamine (Ube et al., 2019b). In wheat, the accumulation of *N*-cinnamoyl-8-oxotryptamine and *N*-cinnamoyl-9-hydroxy-8-oxotryptamine has been shown to act as phytoalexins against pathogen infection caused by *Bipolaris sorokiniana* (Ube et al., 2019a).

During HCAA syntheisis, the condensation of hydroxycinnamoyl-CoA esters and amines is mediated by various hydroxycinnamoyl transferases (HCTs), which catalyze the transfer of hydroxycinnamoyl moieties from CoA esters to acceptor molecules. (Ube et al., 2019b; Zeiss et al., 2021; Liu et al., 2022b). The HCT family includes various isoforms and members with distinct substrate specificities, allowing them to acylate a wide variety of acceptor molecules, such as shikimate, quinate, and other related compounds. This diversity in substrate specificity enables HCTs to participate in different biosynthetic pathways, such as HCAAs, lignins, lignans, and flavonoids, contributing to the complexity and diversity of specialized metabolism in plants.

### 3.2 Terpenes

Terpenes, with over 65,000 known structures, constitute the largest and most diverse class of plant natural products, playing crucial roles in plants, such as defense against herbivores and attraction of pollinators (Schmelz et al., 2014; Zi et al., 2014; Shahi and Mafu, 2021). These compounds are derived from the five-carbon units, isopentenyl diphosphate (IPP) and dimethylallyl diphosphate (DMAPP), generated through the mevalonate (MVA) or the 2-C-methylerythritol-4-phosphate (MEP) pathway (Jacobowitz and Weng, 2020; Ding et al., 2021b). Farnesyl diphosphate (FPP, C15) is typically synthesized via the MVA pathway and serves as the precursor for sesquiterpenes (C15), triterpenes (C30), and sterols. In contrast, within the MEP pathway, IPP and DMAPP, derived from pyruvate and glyceraldehyde-3-phosphate, undergo condensation catalyzed by geranyl diphosphate synthase (GPS) to yield geranyl diphosphate (GPP, C10), serving as the direct precursor for monoterpenes (C10), or by geranylgeranyl diphosphate synthase (GGPPS) to generate geranylgeranyl diphosphate (GGPP, C20), which acts as a precursor for diterpenes (C20) and tetraterpenes (C40) (Jacobowitz and Weng, 2020; Ding et al., 2021b). Terpene synthases (TPSs) catalyze the cyclization of each class-specific building block, acting as gatekeepers in terpenoid production by converting prenyl diphosphates with different chain lengths or distinct cis/trans configurations into diverse terpenoid skeletons (Ding et al., 2021b; Zhan et al., 2022). The P450 enzymes, frequently belonging to the CYP71, CYP76, CYP81, CYP99, and CYP701 families, further enhance the structural complexity and bioactivity of plant terpenoids (Hussain et al., 2018; Ding et al., 2021b).

#### 3.2.1 Monoterpenes and sesquiterpenes

Despite the distinct biosynthetic pathways of monoterpenes and sesquiterpenes, these two classes of compounds collectively contribute to a significant portion of the volatile organic compounds (VOCs) emitted by plants, and have been reported to be involved in plant defense through their pesticidal and antibacterial activity, as well as repellent properties (Lanier et al., 2023). For example, γ-terpinene (monoterpene) exhibits significant antibacterial activity against the rice pathogen *Xanthomonas oryzae* (Yoshitomi et al., 2016); α-pinene (monoterpene) demonstrates toxicity against maize weevil (*Sitophilus zeamais*) (Langsi et al., 2020); α-farnesene (sesquiterpene) acts as an insecticide (Lin et al., 2017), and other monoterpenes such as α-terpinene, *p-*cymene, and β-phellandrene, have been identified as repellent compounds (Bleeker et al., 2009). Furthermore, monoterpenes and sesquiterpenes are frequently utilized by plants to attract pollinators or repel florivores, as exemplified by linalool, limonene, and β-pinene (Boncan et al., 2020; Lanier et al., 2023). In addition, certain non-volatile sesquiterpenes act as phytoalexins, providing direct protection against fungal and bacterial pathogens in plants (Köllner et al., 2013; Schmelz et al., 2014; Ding et al., 2020).

To date, numerous monoterpene synthases and sesquiterpene synthases have been functionally characterized in plants. For instance, in rice, OsTPS24 and OsTPS19 have been identified as monoterpene synthases, producing γ-terpinene and (S)-limonene, respectively (Yoshitomi et al., 2016; Chen et al., 2018). In maize, four monoterpene synthases and thirteen sesquiterpene synthases have been characterized (Block et al., 2019; Saldivar et al., 2023). In tomatoes, TPS5 and TPS39 are involved in the production of the monoterpene linalool (Cao et al., 2014), while TPS9 and TPS12 synthesize several sesquiterpenes, including germacrene C and β-caryophyllene/α-humulene, respectively (Schilmiller et al., 2010). In grapevine (*Vitis vinifera*), specific TPSs, namely VvPNLinNer1, VvPNLinNer2, and VvCSLinNer, have been found to possess the ability to produce linalool (Martin et al., 2010). Indeed, recent studies have provided insights into the synthesis of certain monoterpenes by multi-substrate sesquiterpene synthases in the cytosol (Mercke et al., 2004; Pazouki and Niinemets, 2016). In the case of TPS from cucumber (*Cucumis sativus*), it exhibits C10/C15 multi-substrate characteristic that utilizes GPP as a substrate to produce (E)-β-ocimene, while employing FPP to form (E,E)-α-farnesene (Mercke et al., 2004). This multi-substrate utilization capacity offers an alternative mechanism for regulating the production of monoterpenes and sesquiterpenes by modifying the sizes of different substrate pools in the cytosol, especially under stressful conditions (Pazouki and Niinemets, 2016).

After the initial biosynthesis of terpenes by TPSs, their backbone undergoes various modifications, including oxidation, hydroxylation, or glycosylation. These modifications can lead to the formation of a wide range of structurally diverse terpenoid compounds. A well-studied example is linalool, where CYP76F14 from grapevine catalyzes the oxygenation of linalool, forming (E)-8-carboxylinalool (Bosman and Lashbrooke, 2023). Additionally, CYP76F14 is involved in the synthesis of wine lactone. In another intriguing case, three tandemly duplicated genes of the *CYP71Z* subfamily in maize encode enzymes that catalyze various oxidation reactions on sesquiterpenes, resulting in the formation of zealexin antibiotics (Ding et al., 2020).

#### 3.2.2 Diterpenes and triterpenes

Plants produce a series of diterpenoid compounds, including the widely distributed gibberellin phytohormones and specialized diterpenoids that are exclusively found in specific plant species or families (Hedden and Thomas, 2012; Zerbe and Bohlmann, 2015; Ding et al., 2019). To date, over 7,000 labdane-related diterpenoids have been identified in plants, and they play diverse physiological roles in plant development, defense, and ecological adaptation (Zerbe and Bohlmann, 2015). In angiosperms, the biosynthesis of labdane-related diterpenoids follows a modular process initiated by the carbocation-driven cyclization of the diterpene skeleton through the sequential activity of class II and class I diterpene synthases (di-TPSs) and subsequently enriched by P450-mediated backbone decoration (Ding et al., 2019; Ding et al., 2021b). Firstly, the precursor GGPP undergoes proton-initiated cyclization by class II di-TPSs, resulting in the production of dicyclic *ent*-copalyl diphosphate (*ent*-CPP), (+)-CPP and *syn*-CPP (Ding et al., 2021b). In maize, the class II di-TPSs, ZmAN1 and ZmAN2, are catalytically redundant CPP synthases, with ZmAN1 essential for gibberellin phytohormone biosynthesis, whereas ZmAN2 for the formation of defensive dolabralexin and kauralexin diterpenoids (Mafu et al., 2018; Ding et al., 2019). Other examples of class II di-TPS include maize ZmCPS3 and foxtail millet (*Setaria italica*) SiTPS9 functioning as (+)-CPP synthases, foxtail millet SiTPS6 and rice OsCPS4 acting as *syn*-CPP synthases, and rice OsCPS2 and maize ZmCPS4 serving as *ent*-CPP synthases and 8,13-CPP synthase, respectively (Otomo et al., 2004; Prisic et al., 2004; Murphy et al., 2018; Karunanithi et al., 2020). Subsequently, class I di-TPSs convert these intermediates through ionization-dependent cyclization and rearrangement, leading to the formation of a series of distinct labdane scaffolds (Zerbe and Bohlmann, 2015; Ding et al., 2021b). For instance, ZmKSL2 and ZmKSL4 sequentially convert the *ent*-CPP into *ent*-isokaurene and dolabradiene, respectively (Mafu et al., 2018; Ding et al., 2019). Likewise, OsKSL4 catalyzes the product from OsCPS4, forming the tricyclic momilactone scaffold, while OsKSL7 contributes to the formation of the phytocassane scaffold from the product of OsCPS2 (Otomo et al., 2004). Finally, diterpene backbones are functionalized by other enzyme classes, with the CYP71 clan of cytochrome P450s being the most common, through oxidation and subsequent conjugation processes to enhance their bioactivity (Zerbe and Bohlmann, 2015; Ding et al., 2021b). For example, ZmCYP71Z16 and ZmCYP71Z18 are involved in the oxygenation of *ent*-kaurene, *ent*-isokaurene, and dolabradiene, playing a crucial role in the formation of antibiotics crucial for *Fusarium* stalk rot resistance (Mafu et al., 2018; Ding et al., 2019).

Triterpenoids are also common natural plant defense compounds with potential applications as pesticides, pharmaceuticals, and other high-value products (Singh et al., 2023b). Saponins, for instance, play a key role in promoting plant defense against a wide range of pathogens, insect pests, and herbivores (Hussain et al., 2019). The carbon skeletons of triterpenoids are derived from the common precursor, 2,3-oxidosqualene, through cyclization reactions catalyzed by enzymes such as oxidosqualene cyclases (OSC), including cycloartenol synthases and β-amyrin synthases (Cárdenas et al., 2019). The oxidation of these skeletons is mediated by P450s, contributing to their structural diversity. Subsequent modifications involving UDP-glycosyltransferases (UGTs) and acyltransferases (ATs) further enhance the complexity of triterpenoid structures (Miettinen et al., 2017; Cárdenas et al., 2019).

### 3.3 Alkaloids

Alkaloids are a class of natural nitrogen-containing products, often derived from amino acids such as tyrosine, lysine, ornithine, and phenylalanine (Glenn et al., 2013). Based on their heterocyclic ring system and biosynthetic precursors, alkaloids are classified into diverse categories, including tropane, piperidine, indole, purine, imidazole, pyrrolizidine, isoquinoline, quinolizidine, pyrrolidine, and steroidal alkaloids (Yan et al., 2021). Most alkaloids function as nitrogen storage reservoirs, protective agents against both biotic and abiotic stress, and/or growth regulators (Glenn et al., 2013). For example, α-tomatine, a steroidal alkaloid extracted from various organs of tomato, exhibits antimicrobial and antinutritional activities (You and van Kan, 2021).

Nicotine, the predominant alkaloid found in *Nicotiana* species (Shimasaki et al., 2021). It exhibits strong toxicity and plays a role in plant defense against insects. Additionally, it functions as a potent allelopathic substance, exerting significant growth effects on other plants (Cheng et al., 2021). Nicotine itself comprises heterocyclic pyrrolidine and pyridine rings, with the pyrrolidine ring forming through consecutive reactions catalyzed by Orn decarboxylase (ODC), putrescine N-methyltransferase (PMT), and N-methylputrescine oxidase (MPO), while the pyridine ring results from the involvement of enzymes such as Asp oxidase (AO), quinolinate synthase (QS), and quinolinate phosphoribosyl transferase (QPT) (Kajikawa et al., 2017). The coupling of these two rings is believed to be catalyzed by Berberine Bridge Enzyme-Like Proteins (BBLs) (Kajikawa et al., 2017; Schachtsiek and Stehle, 2019). Recently, CRISPR/Cas editing of genes encoding BBL has been used to obtain nicotine-free non-transgenic tobacco (Schachtsiek and Stehle, 2019).

Another well-known example is Benzoxazinoids (BXs), which are indole alkaloids found in several monocot crop species, such as wheat, maize, and rye (*Secale cereale*) (Ding et al., 2021b; Stahl, 2022). BXs are involved in plant defense against herbivorous arthropods, demonstrating direct insecticidal activity by inhibiting insect digestive proteases through their breakdown products (Zhang et al., 2021). Additionally, BXs play vital roles in plant-microbe interactions and have regulatory effects on various biological processes, including flowering time, auxin metabolism, iron uptake, and potentially aluminum tolerance (Zhou et al., 2018). Given the extensive availability of genetic resources in maize, significant progress in BXs research has been achieved. The core maize BX biosynthesis pathway has been extensively studied and involves seven BX enzymes (BX1–BX5, BX8, and BX9) that catalyze the formation of DIMBOA-Glc from indole-3-glycerol phosphate (IGP) (Meihls et al., 2013; Zhang et al., 2021). These compounds can be further hydroxylated by *O*-methyltransferases (BX10 to BX12) to form 2-hydroxy-4,7-dimethoxy-1,4-benzoxazin-3-one glucoside (HDMBOA-Glc). Moreover, DIMBOA-Glc can be converted to 2,4-dihydroxy-7,8-dimethoxy-1,4-benzoxazin-3-one-O-glucoside (DIM2BOA-Glc) by BX13 and BX7, while DIM2BOA-Glc can be further methylated to form 2-hydroxy-4,7,8-trimethoxy-1,4-benzoxazin-3-one glucoside (HDM2BOA-Glc) by BX14 (Handrick et al., 2016). In rye, the genes *ScBx1*-*ScBx7*, *Scglu*, and *ScGT* have been experimentally confirmed to regulate the majority of BX biosynthesis reactions (Tanwir et al., 2017).

### 3.4 Other specialized metabolites

There is no doubt that numerous other structural types of specialized metabolites exist that may not fit into the categories discussed above. For instance, oxylipins, derived from the oxidation of unsaturated fatty acids such as α-linolenic acid and linoleic acid, play critical roles in plant defense mechanisms (Muñoz and Munné-Bosch, 2020). Plant oxylipins are initiated through enzymatic pathways by 9- and 13-lipoxygenases (LOXs), which oxidize polyunsaturated fatty acids. Among them, the jasmonates (JAs) branch is initiated by 13-lipoxygenase (LOX), leading to the formation of 13-hydroperoxyliolenic acid (13-HPOT), which is further converted to 12-oxo-phytodienoic acid (OPDA) by allene oxide synthase (AOS) and allene oxide cyclase (AOC) (Wasternack and Song, 2017). OPDA is then reduced by OPDA reductase (OPR) and undergoes β-oxidation to generate JA. The JAs are a vital class of plant hormones necessary for regulating plant growth, development, specialized metabolism, defense against insect attack and pathogen infection, and tolerance to abiotic stress. A similar pathway involving 9-LOX activity on linolenic and linoleic acid leads to the 12-OPDA positional isomers, 10-oxo-11-phytoenoic acid (10-OPEA) and 10-oxo-11-phytodienoic acid (10-OPDA), respectively (Christensen et al., 2015). Notably, 10-OPEA exhibits broad toxicity to insects and fungi, likely through the activation of cysteine proteases (Ding et al., 2021b).

Additionally, sulfur-containing metabolites have also been identified in plants. For example, glucosinolates are found in cruciferous plants with defensive roles against insects, (Halkier and Gershenzon, 2006). A recent review has listed up to 137 natural glucosinolates, describing their variability in the R group (Blažević et al., 2020). Moreover, small molecules such as halogenated compounds and peptides also contribute to the formation of numerous functional specialized metabolites (Jacobowitz and Weng, 2020).

## 4 Omics-based approaches for specialized metabolism discovery in plants

**Figure 2:**
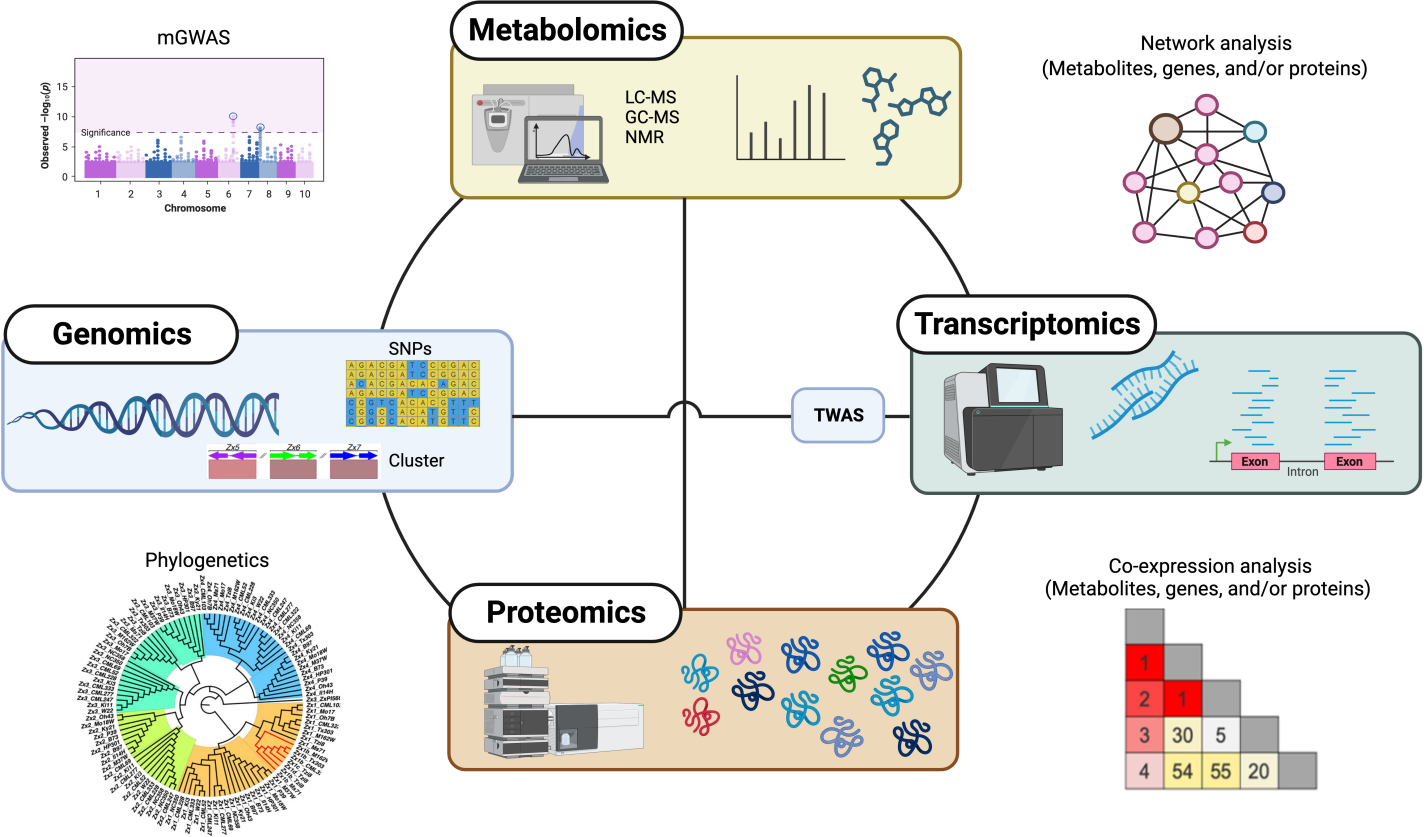
Overview of omics-based approaches for specialized metabolism discovery in plants. Single and combination of omics approaches, including metabolomics, genomics, transcriptomics, and proteomics as well as integrative multi-omics, greatly accelerate the discovery of plant specialized metabolism. mGWAS, metabolite-based genome-wide association analysis; TWAS, transcriptome-wide association analysis.

Although our understanding of the functions of these specialized metabolites is growing, there is still much to explore in terms of biosynthesis and regulation of these natural products, owing to gene and pathway redundancy, the multifunctionality of proteins, or the activity of enzymes with broad substrate specificity (Garagounis et al., 2021; Ding et al., 2021b). In the past decade, omics approaches, such as metabolomics, genomics, transcriptomics, and proteomics, as well as integrative multi-omics approaches, have had an increasing impact on plant specialized metabolism discovery (**Figure 2**), enabling researchers to uncover the intricate mechanisms underlying the biosynthesis, regulation, and biological functions of diverse specialized metabolites in plants.

### 4.1 Metabolomics

Metabolites are often regarded as the bridges between genotypes and phenotypes, and changes in metabolite levels could directly reflect gene function, revealing biochemical and molecular mechanisms underlying phenotypes and facilitating related breeding procedures (Fiehn, 2002). Metabolomics analysis typically relies on a variety of analytical chemistry techniques, such as gas chromatography-mass spectrometry (GC-MS), liquid chromatography-mass spectrometry (LC-MS), and nuclear magnetic resonance (NMR) spectroscopy (Salem et al., 2020). GC-MS is well suited for the identification and quantification of small metabolites with a molecular weight below 650 daltons, which are either volatile metabolites or metabolites easily to volatilize after derivatization, including alcohols, hydroxy acids, fatty acids, and sterols (Ma and Qi, 2021; Ding et al., 2021b). Compared to GC-MS, LC-MS analysis does not require a derivatization step and can measure a broader range of analytes, making it a highly powerful and comprehensive analytical tool. Nowadays, LC-MS has become the most commonly used analytical tool for identifying plant metabolites, including phenylpropanoids, terpenoids, and alkaloids (Lisec et al., 2006; Ma and Qi, 2021). Complementing MS-based analyses, NMR spectroscopy is a fundamental and reliable method for structure elucidation in plant metabolism research, providing valuable insights into the chemical composition and connectivity of plant metabolites (Ma and Qi, 2021). Historically, effectively reducing false-positive peaks, analyzing large-scale metabolic data, and the lack of a comprehensive database for annotating plant metabolites have posed significant challenges in metabolomics.

In recent years, the study of plant metabolites has significantly been supported by the availability of numerous databases, advanced analytical techniques, and computational tools. Databases like NIST, MoNA, and METLIN provide comprehensive resources for accurate and reliable metabolite identification. Meanwhile, the emergence of more sensitive, accurate, and versatile instruments has dramatically improved our ability to identify and quantify low-abundance compounds, even from highly complex mixtures (Fang and Luo, 2019; Jacobowitz and Weng, 2020). In addition, numerous computational tools, such as CANOPUS and GNPS, have been developed, employing MS fragmentation spectra and deep neural networks to accurately assign annotations to unknown metabolites in sample extracts, and construct molecular networks of detected features (Wang et al., 2016; Dührkop et al., 2021; Ma and Qi, 2021). With the continuous advancement in analytical techniques, mass-spectra databases, and computational approaches, metabolomics has emerged as a valuable tool in plant research, providing plant scientists an exceptional opportunity to comprehensively explore specialized metabolism in plants (Yang et al., 2021). The utilization of metabolomics as a tool for monitoring the dynamics of plant metabolites is gaining increasing interest in identifying crucial metabolites associated with tolerance to both biotic and abiotic stresses (Zhang et al., 2017; Christ et al., 2018; Billet et al., 2020). For instance, UPLC-DAD-MS-based metabolomics enabled the analysis of downy mildew symptomatic grapes leaves, revealing certain stilbenoids as significant biomarkers of the infection (Billet et al., 2020). Similarly, utilizing UPLC-QTOF to assess the effects of low nitrogen stress on wheat flag leaves during two crucial growth periods, the study revealed that flavonoids likely serve as biomarkers of low nitrogen stress (Zhang et al., 2017).

Other new technologies, such as flavoromics, have been also developed to study specific groups of metabolites. Metabolomics utilizes both targeted and untargeted methodologies to identify and characterize a diverse range of small molecule metabolites. In contrast, flavoromics is specialized in pinpointing metabolic components directly linked to flavors. Flavoromics represents an extensive interdisciplinary domain that integrates analytical chemistry, bioinformatics, and sensory science. Its primary aim is to comprehensively explore flavor compounds found in various substances, particularly in food and beverages. This field encompasses intricate processes involved in the identification, quantification, and understanding of the complex composition of both volatile and non-volatile compounds that influence sensory perceptions associated with taste and aroma (Pérez-Jiménez et al., 2021; Keawkim and Na Jom, 2022).

### 4.2 Genomics

With the increasing speed and decreasing costs of sequencing and genome assembly platforms, a large number of high-quality plant genomes have been assembled and released (Kress et al., 2022), providing a powerful foundation for studying plant specialized metabolism. Unlike metabolic pathway genes forming biosynthetic gene clusters (BGCs) in prokaryotes, genes involved in plant specialized metabolism are often randomly distributed across the plant genome. However, studies have revealed the existence of operon-like clusters of specialized metabolic pathway genes in plants, providing a strategy to identify genes involved in plant specialized metabolism in the post-genomic era (Jacobowitz and Weng, 2020; Zhan et al., 2022). To date, the majority of plant BGC-encoded products that have been characterized demonstrate activity against a wide range of pests, pathogens, and competing plants (Polturak and Osbourn, 2021).

Phylogenetic analysis can offer valuable insights to enhance the prioritization of candidate genes. The combined use of genomic sequence and phylogenetic-based gene discovery has been successfully applied to identify genes involved in plant specialized metabolism, such as terpenoid metabolism. In the study on the foxtail millet *TPS* gene family, a total of 39 genes were identified by mining available genomic data using the BLAST against a curated protein database of known plant TPSs, with 32 of these genes having full-length sequences. Next, functional classification of these *TPS* genes was conducted through analysis of signature sequence motifs and phylogenetic analysis to further narrow down the number of candidates, revealing that SiTPS6, SiTPS9, SiTPS34, and SiTPS35 belong to class II di-TPS enzymes, SiTPS28 and SiTPS29 show similarity to *ent*-kaurene synthase activity, and SiTPS5, SiTPS8, and SiTPS13 are closely related to class I di-TPSs (Karunanithi et al., 2020). Similarly, in the bioenergy crop switchgrass (*Panicum virgatum*), mining of genome and transcriptome inventories suggested a large *TPS* gene family with over 70 members, consisting of 44 mono- and sesqui-*TPS* genes and 30 di-*TPS* genes, and phylogenetic analyses confirmed that 35 of these members belong to the TPS type-a clade (Muchlinski et al., 2019). Such approaches have also been applied in studying P450-catalyzed biosynthesis of furanoditerpenoids in switchgrass. Through systematic phylogenetic analysis of the switchgrass P450 CYP71Z subfamily gene, CYP71Z25-CYP71Z29 were identified as candidate enzymes for subsequent biochemical analysis (Muchlinski et al., 2021).

### 4.3 Transcriptomics

Transcriptomics provides direct insights into real-time gene expression profiles and is one of the most commonly used types of omics. RNA sequencing (RNA-Seq) has emerged as a powerful and effective method for conducting large-scale transcriptomic research, particularly in most non-model plants that lack a high-quality reference genome (Yang et al., 2021; Wang and Huo, 2022). The expression of functionally related genes involved in specialized metabolic pathways is often highly correlated in spatial and temporal dimensions (Schmelz et al., 2014; Ding et al., 2020). Therefore, gene expression can facilitate the discovery of metabolic pathways by mining organ-specific genes, gene expression clusters, and performing coexpression analysis. Transcriptional coexpression analysis, which is based on the premise that a set of genes involved in a biological process are co-regulated and co-expressed under given conditions, has been successfully employed to identify genes involved in plant specialized metabolism, such as terpenoids, glucosides, benzoxazinoids, flavonoids and others (Ding et al., 2021b). For example, gene coexpression analysis identified three CYP71 family P450s in maize terpenoid biosynthesis, which were not identified by extensive forward genetic studies (Ding et al., 2021b). To accurately measure the relationship among genes, an unbiased RNAseq database is essential. With increasingly affordable next-generation sequencing technologies, large-scale transcriptomic datasets are routinely generated and are becoming publicly available. Various statistical correlation-based approaches are used for coexpression analysis, such as Spearman Correlation Coefficient (SCC) and Pearson Correlation Coefficient (PCC). Mutual Rank (MR), the geometric mean of the ranked PCCs between two genes, has been used to measure gene coexpression (Poretsky and Huffaker, 2020). When using coexpression analysis to identify unknown biosynthetic genes in a target pathway, a key bait gene with a known function is often required for the analysis (Singh et al., 2022). The cutoff scores used to identify candidate pathway genes or construct coexpression networks are often selected arbitrarily.

Additionally, coexpression analysis plays a unique role in identifying non-enzymatic components, such as transcription factors and transporters, which are crucial for the efficient functioning of metabolic pathways. In the context of investigating the molecular mechanisms underlying apple (Malus × domestica) color formation, the utilization of pairwise comparisons and weighted gene coexpression network analysis (WGCNA) led to the identification of *MdMYB28* as a key regulatory gene that negatively regulates anthocyanin biosynthesis (Ding et al., 2021a). Similarly, employing the same method, a pepper MYB transcription factor, CaMYB48, was identified as a critical regulatory component in capsaicinoid biosynthesis (Sun et al., 2020).

Successful coexpression analysis depends on the correlation of biosynthetic genes with their respective metabolites *in planta*. This approach will not be useful in some cases if the site of biosynthesis is different from the site of metabolite accumulation. Also, this approach may not be applicable in situations where biosynthetic intermediates are produced in one part of the plant and then transported to another part, where biosynthesis is completed.

As multicellular organisms, plants have evolved different cell types for cellular responses uniquely to different environmental cues. Single-cell sequencing technologies are being employed to explore cell-type-specific responses to stresses in plants (Cole et al., 2021). In addition to elucidating the spatiotemporal distribution of metabolic pathways at single-cell resolution, these technologies offer a valuable strategy for identifying candidate pathway genes. For example, Sun et al. utilized single-cell RNA sequencing to localize the transcripts of 20 MIA (monoterpenoid indole alkaloids) genes in different cell compartments and predicted several candidate transporters likely involved in shuttling MIA intermediates between inter- and intracellular compartments (Sun et al., 2023).

### 4.4 Proteomics

The development of high-quality sequenced genomes enables proteomics to effectively facilitate the prioritization of candidate biosynthetic enzymes in plant specialized metabolic pathways (Ding et al., 2021b). High-throughput protein sequencing technology includes iTRAQ (isobaric tags for relative and absolute quantification) and DIA (data-independent acquisition). Recent advances in mass spectrometry (MS)-based proteomics technologies have enabled the comprehensive identification, quantification, validation, and characterization of a diverse range of proteins in specific organs, tissues, and cells (Champagne and Boutry, 2016). For example, untargeted proteomics using data-dependent acquisition (DDA) with a quadrupole time-of-flight (Q-TOF) tandem mass spectrometer allows the quantification of thousands of detectable proteins in samples (Hart-Smith et al., 2017). A comparative proteomic analysis using mass spectrometry (MALDI-TOF/TOF) was conducted on resistant cotton (*Gossypium barbadense*) infected with *Verticillium dahliae*, revealing 188 differentially expressed proteins and identifying several genes involved in secondary metabolism, reactive oxygen burst, and phytohormone signaling pathways (Gao et al., 2013). However, owing to higher costs and lower sensitivity, proteomics is being utilized less frequently than other omics techniques for metabolic pathway gene discovery.

### 4.5 Integrative multi-omics approaches

Metabolites are interconnected and form a complex and tightly regulated metabolic network, making the use of a single-omics technique prone to inherent biases. With technological advances in profiling metabolites, genes, and proteins, the application of combined multi-omics technologies provides new strategies and opportunities to discover stress-related metabolic pathways in plants.

Metabolite-based genome-wide association studies (mGWASs), which make use of both genomics and metabolomics data, have emerged as a powerful tool for linking metabolites with biosynthetic and regulatory genes (Fang and Luo, 2019; Ding et al., 2021b). mGWASs greatly facilitate large-scale gene–metabolite annotation and identification in plants, offering valuable insights into the genetic and biochemical basis of the plant metabolome. For example, mGWASs have been successfully performed to identify biosynthetic genes involved in maize specialized metabolisms, such as benzoxazinoids, terpenoids, and flavonoids (Zhou et al., 2019; Ding et al., 2021b; Förster et al., 2022). For mGWASs, increasing the number and diversity of accessions in the panel is prioritized over having multiple replicates of the same accession since a larger diversity panel can provide a broader representation of genetic variation and increase the power to identify significant associations between metabolites and genes across different accessions (Zhou et al., 2019).

In addition to mGWASs, metabolite-based quantitative trait locus analysis (mQTL) based on bi-parental populations has also been employed for pathway gene discovery in plants. For instance, mQTL analysis was performed and successfully identified three P450s, ZmCYP81A37, ZmCYP81A38, and ZmCYP81A39, for the biosynthesis of sesquiterpenoid antibiotics zealexins in maize (Ding et al., 2020). mQTL and mGWAS are two complementary forward genetic approaches, and their combination provides effective information for candidate gene mining. These metabolite-based genetic mapping approaches also complement other methods in metabolite identification, including coelution tests with known compounds and feature network analysis.

Using metabolite concentration ratios (metabolite ratios) as mapping traits in mGWASs has been found to reduce overall biological variability in population datasets and improve statistical associations (Petersen et al., 2012). The nature of a metabolite ratio may directly reflect the biochemical function of an enzyme or transporter associated with the pair of metabolites. This approach is particularly useful when prior knowledge of the biosynthetic pathway is available. By employing metabolite ratios as traits in mGWASs, researchers have successfully identified biosynthetic genes involved in plant specialized metabolism. For example, in a maize flavonoid biosynthesis study, an additional FOMT (flavonoid *O*-methyltransferase)-encoding gene was identified by an mGWAS using the apigenin/genkwanin ratio as a trait. This gene was not detected by mGWASs directly using the concentrations of either apigenin or genkwanin (Förster et al., 2022).

Due to linkage disequilibrium (LD), genetic markers (e.g., SNPs) identified by mGWASs often reside outside the candidate genes and can sometimes be relatively far away from them, making it challenging to select the candidate genes. Transcriptomics, in combination with mGWASs, offers an efficient approach to prioritize the candidate genes at mGWAS loci. For example, we recently used this approach to prioritize a reductase catalyzing A-series kauralexin biosynthesis at an mGWAS locus, which spans ~800 kb containing 58 predicted genes (Ding et al., 2019). In addition, transcriptome-wide association studies (TWASs) in combination with mGWASs have been proven to be very helpful in prioritizing causal genes at mGWAS loci in humans (Ndungu et al., 2020). Its potential in prioritizing candidate biosynthetic genes in plants is also promising.

In addition to the integration of omics approaches discussed above, other integrative multi-omics analyses are also highly valuable in discovering plant specialized metabolism. For example, the mechanism of light-induced anthocyanin biosynthesis in eggplant was analyzed using a combination of transcriptomics and proteomics, revealing a regulatory model for light-induced anthocyanin biosynthesis (Li et al., 2017). Moreover, the integration analysis of transcriptomics and metabolomics data enables mutual validation, facilitates the discovery of key genes, metabolites, and metabolic pathways from extensive datasets, and provides a comprehensive understanding of complex biological processes.

Single-cell transcriptomics and single-cell metabolomics are also valuable tools in the study of plant specialized metabolism. These techniques allow researchers to examine the molecular profiles of individual cells, providing insights into cellular heterogeneity and revealing rare or transient metabolic states that might be overlooked in bulk analyses (Vandereyken et al., 2023). For example, the combination of single-cell transcriptomics and single-cell metabolomics allowed the identification of a reductase for anhydrovinblastine biosynthesis in the MIA pathway (Li et al., 2023).

Collective analyses of the transcriptome, proteome, and metabolome can uncover metabolic pathway inter-conversions and drive gene discoveries in plants, by associating temporal and spatial expression levels of genes and enzymes with metabolite abundance across different samples. (Ding et al., 2021b). For example, a time-course experiment was conducted on maize stem tissues to study zealexin biosynthesis in response to fungal elicitors, and the data clearly showed that genes, enzymes, and metabolites involved in the zealexin pathway had a similar expression pattern (Ding et al., 2020), providing a valuable strategy for studying plant specialized metabolism.

Integrative multi-omics approaches hold great promise for advancing our understanding of plant specialized metabolism. By combining data from various omics techniques, researchers can overcome individual technique limitations, gain a more holistic view of metabolic networks, and identify key genes and metabolic pathways involved in plant stress responses.

## 5 Functional validation of candidate pathway genes

**Figure 3:**
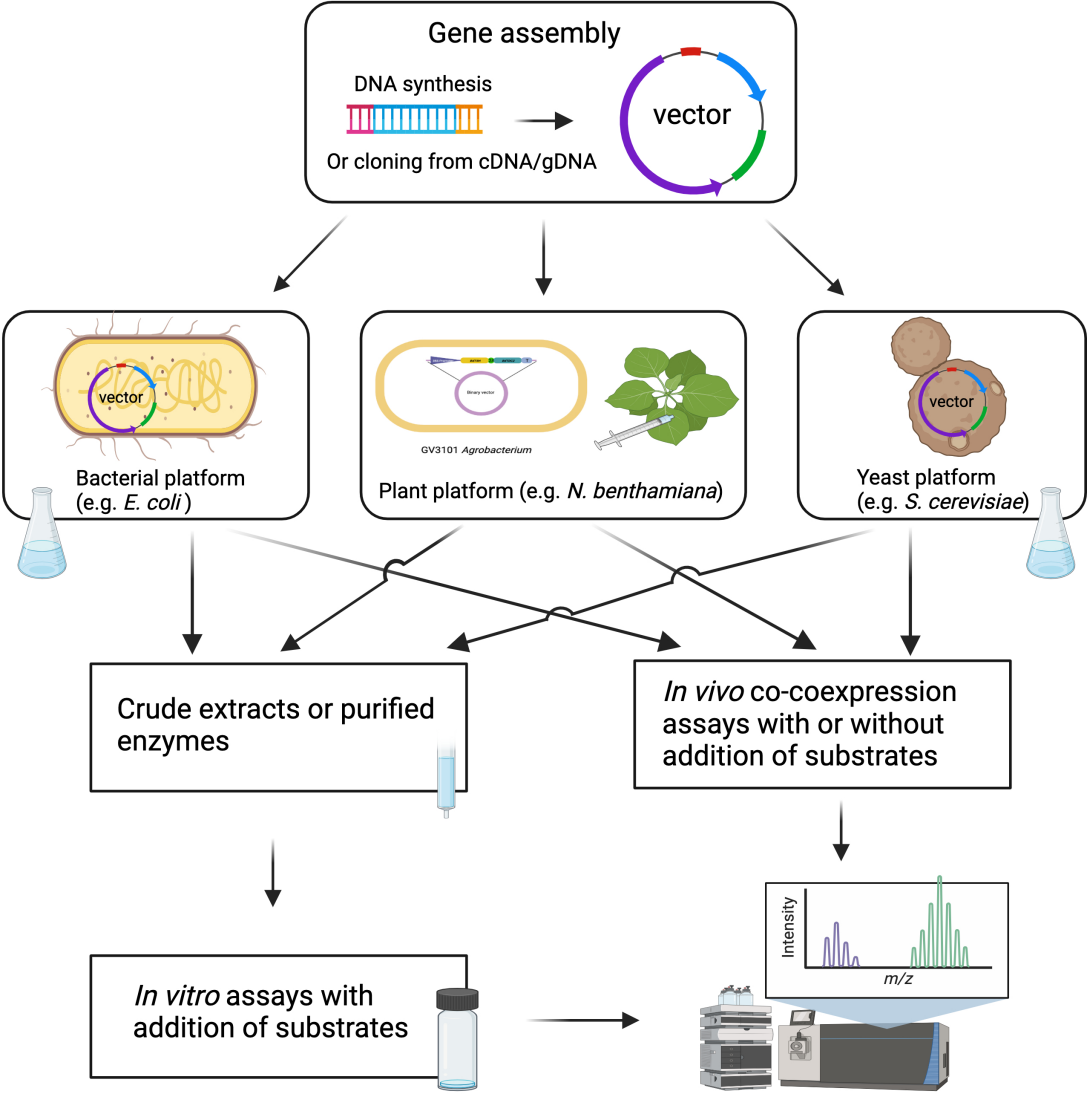
Schematic overview of high throughput approaches for characterization of candidate biosynthetic genes. The figure was created with BioRender.com.

Following candidate gene identification, the verification of enzyme function requires robust biochemical and genetic approaches. Compared to traditional molecular cloning, which requires a considerable amount of time and human resources, DNA synthesis is becoming a cost-effective approach for the rapid assembly of candidate genes into expression vectors for functional analysis (Blaby and Cheng, 2020). DNA synthesis, along with synthetic biology and genetic engineering tools, allows for larger-scale enzyme biochemical analyses and metabolic pathway reconstruction in heterologous hosts like yeast, *E. coli*, and *N. benthamiana* (**Figure 3**). Biochemical approaches for functional validation may face challenges such as low protein expression, low enzymatic activity, and requirements for co-enzymes and substrates. To overcome these issues, *in vivo* expression systems through combinatorial enzyme expression in microorganisms and plants have been developed. Among them, *Agrobacterium*-mediated transient expression in *N. benthamiana* has become a routine system for plant specialized metabolism research (Bach et al., 2014; Tiedge et al., 2020). This plant expression system has expanded our understanding of biosynthetic pathways, facilitated the identification of novel enzymes, and provided a platform for efficient production of valuable metabolites. This system offers several advantages, including the ease of coexpressing multiple genes in a combinatorial manner, the presence of endogenous biosynthetic pathway precursors, and the ability to interrogate enzyme activity without the need for protein purification (Ding et al., 2021b). Recent advances in specialized metabolism discovery using this approach include the demonstration of the 10-gene maize zealexin pathway, the large-scale production of rice momilactones, and other valuable plant natural products (Ding et al., 2019; Ding et al., 2020; De La Peña and Sattely, 2021). Despite the benefits of *N. benthamiana* as an expression system, the presence of endogenous enzymes and similar pathways in this plant species could potentially interfere with introduced pathways. For example, endogenous glycosyltransferases in *N. benthamiana* could derivatize the early MIA pathway intermediates, and the removal of these endogenous enzymes could facilitate the production of the early MIA pathway product, strictosidine, in *N. benthamiana* (Dudley et al., 2022).

Coexpression of multiple genes using the *Agrobacterium*-mediated transient expression system in *N. benthamiana* is typically accomplished by co-infiltration of multiple *Agrobacterium* strains that each contains one target gene. To improve the efficiency of co-expressing multiple genes, researchers have explored the use of 2A peptides, which enable the expression of multiple proteins under the control of a single promoter (Sharma et al., 2012; Liu et al., 2017). For example, the F2A peptide was successfully used to express three betalain biosynthetic genes under the control of Cauliflower Mosaic Virus (CaMV) 35S promoter in *Arabidopsis* (He et al., 2020). Potentially, 2A-containing peptides could be utilized to co-express multiple pathway genes in the *Agrobacterium*-mediated transient expression system, enhancing the likelihood of plant cells co-expressing multiple biosynthetic genes to increase the production of target metabolites while reducing the formation of intermediate metabolites.

Gene function can also be validated by using genetic mutants obtained through various methods, including genome-wide variation mining, classical ethyl methane sulfonate-induced mutations, T-DNA insertion lines, or expanding transposon-insertion mutant collections (Ding et al., 2021b). For plant species with available genetic resources, these mutant lines can be valuable tools to study the effects of gene disruption on specialized metabolism and the resulting phenotypes. To precisely create mutations in candidate pathway genes, CRISPR/Cas9 genome editing approaches and RNA-guided gene silencing techniques are commonly used in plant research. These tools allow researchers to create stable and transient gene modifications for functional studies (Mei and Whitham, 2018; Zhu et al., 2020). For example, we recently developed a maize *zx1 zx2 zx3 zx4* quadruple mutant using a CRISPR/Cas9 approach, which lacks zealexin production and has a changed root microbiome (Ding et al., 2020). The combination of biochemical and genetic approaches, along with advancements in DNA synthesis, synthetic biology, and gene editing technologies, has significantly enhanced our ability to validate the function of candidate pathway genes in specialized metabolism. In addition, cell-free systems have been used to characterize candidate pathway genes and study complex, modular pathways of plant specialized metabolism *in vitro* (Tiedge et al., 2020). These tools and techniques discussed here will continue to play a vital role in advancing our understanding of plant stress-related specialized metabolism and in harnessing these specialized pathways for improving plant stress resilience.

## 6 Conclusion and future perspectives

The advancements in genomics, metabolomics, transcriptomics, and proteomics, as well as integrative multi-omics, have significantly enhanced our understanding of specialized metabolism in plants (Singh et al., 2022). Other omics, such as flavoromics and lipidomics, also contribute to the study of plant specialized metabolites. These approaches have paved the way for studying pathway genes and their biological functions more efficiently, leading to a better understanding of the production of specialized metabolites and their roles in plant stress responses. Additionally, with the continuous improvements in high-throughput metabolic profiling and sequencing technologies, mGWAS has become a potent forward genetics strategy to unravel the genetic and biochemical basis of specialized metabolism in plants. Moreover, genetic engineering and synthetic biology offer exciting possibilities for developing plants with modified metabolic traits. By manipulating or introducing novel metabolic pathways, scientists can create plants with enhanced stress resilience and other desirable traits in the coming years. Techniques like CRISPR/Cas9 have revolutionized gene editing and made it easier to engineer specific traits in plants.

The integration of multi-omics approaches, such as combining data from genomics, metabolomics, transcriptomics, and proteomics, will be crucial in furthering our understanding of plant specialized metabolism. These data-driven approaches, coupled with advanced computational methods, biochemical techniques, synthetic biology, and genetic approaches, can provide valuable insights into complex metabolic and biological processes. Additionally, the development of efficient plant transformation methods will play a vital role in applying the knowledge gained from specialized metabolism research to crop improvement. Faster and more reliable transformation techniques will enable the practical implementation of genetically modified plants with desired traits, such as stress tolerance.

The future of specialized metabolism research in plants looks promising and will be driven by advances highlighted in this review. By leveraging the knowledge obtained through omics-based approaches and genetic engineering as well as other techniques, we expect to see the emergence of more stress-resistant plants with modified metabolic traits, which will contribute to sustainable agriculture and global food security in the future.

## Author contributions

MW: Writing – original draft, Writing – review & editing. TN: Writing – review & editing. YD: Writing – review & editing, Writing – original draft.
